# Combined Gold Recovery and Nanoparticle Synthesis in Microbial Systems Using Fractional Factorial Design

**DOI:** 10.3390/nano13010083

**Published:** 2022-12-24

**Authors:** Suanny Mosquera-Romero, Juan Anaya-Garzon, Cristina Garcia-Timermans, Jo Van Dorpe, Anne Hoorens, Nadine Commenges-Bernole, Kim Verbeken, Korneel Rabaey, Jeet Varia

**Affiliations:** 1 Center for Microbial Ecology & Technology (CMET), Ghent University, Coupure Links 653, B-9000 Gent, Belgium; 2 ESPOL Polytechnic University, Escuela Superior Politécnica del Litoral, ESPOL, Facultad de Ciencias Naturales y Matemáticas, Campus Gustavo Galindo km. 30.5 Vía Perimetral, Guayaquil P.O. Box 09-01-5863, Ecuador; 3 University Grenoble Alpes, University Savoie Mont Blanc, CNRS, Grenoble INP (Institute of Engineering and Management University Grenoble Alpes), LEPMI, 38000 Grenoble, France; 4 Department of Pathology, Ghent University Hospital, Entrance 23, Corneel Heymanslaan 10, B-9000 Ghent, Belgium; 5 Department of Materials, Textiles and Chemical Engineering, Ghent University, Technologiepark-Zwijnaarde 46, B-9052 Gent, Belgium; 6 Centre for Advanced Process Technology for Urban Resource Recovery (CAPTURE), Frieda Saeysstraat 1, B-9000 Ghent, Belgium

**Keywords:** metal recovery, bio-precipitation, hydrogen electron-donor, metallic nanoparticles

## Abstract

Green synthesis of gold nanoparticles (AuNPs) using microorganisms has been generally studied aiming for high-yield production and morphologies appropriated for various applications, such as bioremediation, (bio)sensors, and (bio)catalysis. Numerous approaches showed the individual effect of factors influencing the synthesis of AuNPs with limited analysis of the governing factors enhancing the production and desired quality of the precipitates. This study proposes a fractional-factorial design to investigate the simultaneous influence of seven environmental factors (cell concentration, temperature, anoxic/oxic conditions, pH, gold concentration, electron donor type, and bacterial species) on the recovery yield and synthesis of targeted AuNPs. Various sizes and morphologies of the AuNPs were obtained by varying the environmental factors studied. The factors with significant effects (i.e., 0.2 mM Au and pH 5) were selected according to statistical analysis for optimal removal of 88.2 ± 3.5% of gold and with the production of valuable 50 nm AuNPs, which are known for their enhanced sensitivity. Implications of the cytochrome-C on the bacterial mechanisms and the provision of electron donors via an electrochemical system are further discussed. This study helps develop gold recovery and nanoparticle synthesis methods, focusing on the determining factor(s) for efficient, low-cost, green synthesis of valuable materials.

## 1. Introduction

Functional materials for diverse technological domains can be obtained by synthesizing nanostructures for their direct use or by doping existing materials to enhance their current properties. To illustrate some cases, Pd-based nanoparticles have been used for bioremediation of micro-pollutants in wastewater [1,2], supercapacitors with highly efficient charge transport and excellent mechanical stability have been obtained using Co-based nanoparticles [3,4,5] and enhanced elastic properties of materials were obtained by the inclusion of carbon-based nanocomposites [6,7,8].

Nanomaterials of noble metals, such as gold nanoparticles (AuNPs), are of particular interest as their chemical, electronic, and optical properties are prominent for diverse applications, including (bio)catalysis, analytic upgrade detection, medical diagnostics, and environmental remediation. Considering the high value of AuNPs, recovery of gold and AuNPs synthesis from primary or secondary sources makes sense for circular economy approaches. For instance, AuNPs can be obtained by treating waste streams such as jewelry leachate, eluates from activated carbon, or spent liqueurs, with gold concentrations ranging from 0.005–10 mM [9,10,11]. 

Continuous efforts have been devoted to developing high-yield, low-cost, low-energy, and harsh chemical-free methods for AuNPs production [12,13]. Classical chemical, physical and physicochemical methods to synthesize functional metallic nanoparticles are commonly employed, yet those processes are considered chemical- and energy-intensive [14,15]. The application of microorganisms as potential ‘nano-factories’ has gained momentum for the biosynthesis of functional metallic nanoparticles (NPs) [16,17], with properties similar to classically synthesized NPs [18,19]. The progress of these green approaches requires efficiently establishing optimal environmental conditions to trigger the production of the targeted nanomaterials.

Microbes employ various mechanisms during the process of AuNPs synthesis. Those mechanisms are passive biosorption, physicochemical reduction, and active bacterial metabolism (e.g., dissimilative or resistance) [16,20]. Biosorption includes processes mainly associated with the cell wall [20] and extracellular polymeric substances (EPS) [21]. The initial pH can control NPs formation [22] since it affects the solubility and speciation of metal ions, the charge of EPS, and (de)protonation of the cell wall [23,24]. Then, the metallic reduction is facilitated by microbial oxide-reductase enzymes [16,20,25]. Cytochrome-c and hydrogenase enzymes support bacterial growth and are speculated to be involved in the electron transfer to metal ions [26]. EPS secreted during bacterial stress could also act as a reducing agent for Au^3+^ and form nucleation sites [23]. Finally, the cell wall lipid structures are employed as capping agents to synthesize stable AuNPs [24]. Considering the various microbial mechanisms mentioned, NPs can be located extra/intracellularly or on the cell wall [25]. The microbially mediated method is an eco-friendly and cost-effective alternative due to the self-production of all chemical agents needed to synthesize NPs under ambient conditions.

Active microbe–metal interactions with initially viable cells contribute to metal recovery and AuNPs synthesis. For instance, the *Shewanella* genus is recognized for its various respiratory strategies [27], leading to metal recovery and AuNPs synthesis [18,28]. *Cupriavidus metallidurans* CH34 is recognized as a model species for high resistance to heavy metals such as Co, Zn, and Cd [29]. *Cupriavidus* strains synthesized gold into AuNPs [30] by detoxifying Au(III)-complexes via an energy-dependent efflux, as well as intra or extracellular reductive precipitation [30]. 

A notable limitation of the active-microbial processes is the necessity of an electron donor [21]. Various reports are available on microbial metal precipitation via the use of lactate [17,26] and H_2_ [22,28,31,32], from which H_2_ is especially highlighted as a clean energy vector [33]. Nevertheless, its supply from pressurized containers in scaled-up processes is limited by the high cost of production, transport, storage, and supply [34]. Alternatively, H_2_ generated in electrochemical systems can supply the electron donor for the biogenic NPs formation at the cathodic chamber [21] or by harvesting the H_2_ gas outside the electrochemical reactor [35]. The combination of electrochemical systems with microbially mediated nanoparticle synthesis is a research domain of growing interest due to the potentially better-controlled in situ supply of electron donors [1,21]. 

This research investigates the metal sorption, reduction, and precipitation of AuNPs by a fractional factorial design that simultaneously analyzed seven operational factors (Table 1), chosen according to what presumably influences gold recovery and AuNPs size. Other reports have only presented the influence of much fewer factors individually, with one response variable during bacterial-mediated AuNPs [9,18,22,28]; see also Table S1. Just very recently, the fractional factorial design has been employed to investigate the determining factor for green synthesis of AuNPs by employing coffee extract [36], tuber extracts [37], and upland cress [38]. However, to our knowledge, the investigation of initially active bacterial cells of two species for AuNPs synthesis in a fractional factorial design has not been reported so far. This report explores statistical analysis for the design of experiments, and also invests in the inferences of the mechanism for AuNPs formation with a final suggestion for affordable electron provision with electrochemical systems.

## 2. Materials and Methods

### 2.1. Gold Solutions and Bacterial Cultivation

Gold chloride trihydrate (HAuCl_4_, Sigma-Aldrich, Bornem, Belgium) was dissolved in deionized water (Merck Millipore, Burlington, MA, USA) to prepare a stock solution (5 g L^−1^). The experimental solutions were prepared from the stock by dissolving in 0.9% *w*/*v* NaCl (Carl Roth, Karlsruhe, Germany).

Frozen cells (20% glycerol at −80 °C) of *S. oneidensis* MR-1 (LMG 19005) and *C. metallidurans* CH34 (SCK-CEN, Mol, Belgium) were inoculated under aerobic conditions on tryptic-soy agar (Oxoid, England) at 28 °C. Individual colonies were inoculated in tryptic-soy broth (Carl Roth, Germany) aerobically at 28 °C agitated in a rotatory shaker (KS 400i, IKA, Staufen, Germany) set at 120 rpm. Cells were harvested at the stationary phase (after 72-h of incubation at an optical density, OD_610_, of ≈1) by centrifugation (10 min at 10,000 rcf, Sorval RC6+, Thermo Scientific, Waltham, MA, USA), washed twice with sterile 0.9% *w*/*v* NaCl, and finally concentrated to a value of OD_610_ ≈ 7 [32]. Concentrated cell stocks were made anoxic by degassing for 21 cycles of vacuum and filling with N_2_ gas (Linde, Munich, Germany). A control was set with non-viable cells for experimental validation by autoclaving the viable cell stocks for 21 min at 121 °C. 

### 2.2. Microbial-Mediated Gold Recovery

Microbe-mediated recovery was studied by applying a design of experiments (DOE) of a fractional-factorial matrix (Resolution III), which is ideal for fast-track screening [39]. These consisted of 7 factors at 2 levels, selected based on previous studies [24,25,30], and arranged in an orthogonal matrix. Further details about the selection of the levels of each factor can be seen in Supplementary Data. The standard order was randomized to ensure that each trial was independently distributed to eliminate any trial-combinations bias. The resulting experimental order (Table 1) is used herein as the main label of each unit (e.g., R7 corresponds to the seventh experimental order).

Experiments were conducted for 72 h in serum bottles (120 mL) filled with 50 mL of the metal solution at specified metal concentration and pH (Table 1). Then, the bottle was plugged with butyl rubber stoppers and sealed with an aluminum crimp. Reactors were autoclaved at 121 °C for 21 min without observing any precipitation after autoclaving. The headspace of the serum bottle in anoxic conditions was set with N_2_ (Linde, Germany) by degassing for 21 cycles of vacuum and filling with the gas. Electron donors were supplied either to the solution (sodium lactate) or in the headspace (H_2_ gas), and cells were inoculated from the concentrated stocks, according to Table 1. The reactors were placed on incubators at the corresponding temperature with a rotatory shaker (KS 400i, IKA, Germany) set at 120 rpm. 

Reactor conditions that showed maximized metal recovery were replicated in triplicate. An abiotic control was set without the inoculation of the bacterium species, and another control with inactivated cells.

### 2.3. Analytical Methods

Gold concentrations were measured with inductively coupled plasma optical emission spectrometry (ICP-OES) (Varian Vista-MPX CCD simultaneous, Mulgrave, Australia). Samples were filtered (0.2 µm) and diluted with 1% *v/v* HNO_3_ (65% A.R. grade, Chem-Lab, Zedelgem, Belgium) prior to analysis. This approach allowed quantifying the Au ions remaining in the solution (i.e., unrecovered).

The headspace pressure in the serum reactor was measured with a tensiometer (Infield7, UMS, Regstrup, Denmark). A compact gas chromatograph (GC, Global Analyzer Solutions, Breda, The Netherlands) equipped with a pre-column (Molsieve 5A) and a column (PorabondQ), analyzed the gas composition at the beginning and end of the experiments. Equation (1) calculated the available concentration of H_2_ in the aqueous phase (C_H2_) based on Henry’s constant (H^cp^) based on the partial pressure (P_H2_).

(1) $$C_{H_{2}}=P_{H_{2}}\times H^{cp}\;$$

Concentrated bacterial stocks and reactor samples were analyzed using flow cytometry (FC) to obtain cell numbers and viability based on membrane integrity [40]. Samples diluted in freshly filtered NaCl 0.9% *w/v* were stained with SYBR^®^ Green I (100× concentrate in 0.22 μm-filtered dimethyl sulfoxide, Invitrogen) to obtain the total cell count. The SYBR^®^ Green was combined with propidium iodide (SYBR^®^ Green and 50 × 20 mM PI in 0.22 μm-filtered dimethyl sulfoxide) for viability analysis. The PI only enters cells with a damaged or permeable membrane, differentiating intact versus putatively dead or damaged cells. Stained samples were incubated for 13 min at 37 °C to make FC measurements using the Accuri C6+. The occurrence of cells was determined by analyzing the events per volume on green vs. red fluorescence plot in the BD CSampler Plus software (version 1.0.264.21, BD Biosciences, Erembodegem, Belgium) on gating defined by the density plots. The bacteria viability ratio was obtained by the quantification of cell densities in the different fluorescent channels.

### 2.4. Characterization of NPs

Raman spectroscopy can be applied to identify the (bio)molecules expressed to assess the phenotype present in bacteria [41]. AuNPs can amplify the Raman signal of biochemical components in their close vicinity (hundred-nm range), in the effect known as SERS [42]. Therefore, this technique was used to study which molecules could be involved as nucleation sites for the reduction of ionic Au^3+^ and synthesis of AuNPs. Raman was analyzed in the WITec Alpha300R+ spectroscope (Toptica, Ulm, Germany) with the sampling treatment as previously detailed [41]. A 5 µL drop was allowed to dry until evaporation in a CaF_2_ slide (grade 13 mm diameter by 0.5 mm polished disc, Crystran Ltd., BOP, UK) before Silica gel was measured as a control for the instrument performance. Bacterial samples were measured with a grating of 300 g mm^−1^. The metadata are available in the Supplementary Data.

Spectroscopic and electron microscopy techniques were used to identify, locate, and characterize AuNPs. UV-vis spectra scans of metallic solutions carried on the light wave spectrophotometer (Biochrom WPA1100nm II, CAM, UK) identified characteristic adsorption peaks that would indicate any nano-to-microscale precipitate [43]. 

Transmission electron microscopy (TEM) imagery was obtained by a Zeiss TEM 900 (Carl Zeiss, Oberkochen, Germany). The protocol is detailed in the Supplementary Data. The dimensions (width and length) of the bacteria cells and the average of 10 identified nanoscale precipitates were determined using ImageJ software (Windows softwater, IE 6.0, Microsoft Java 1.1.4) [44]. For SEM determination in a Quanta-450F field emission gun (FEG) (Thermo Scientific, USA), 200 µL of filtered samples (0.2 µm) were placed on a sample holder with carbon tape and dried at room temperature before analysis. Energy-dispersive X-ray spectroscopy (EDX) was used to determine the elemental compositions at the same SEM analysis point.

### 2.5. Data Analysis

An essential indicator for metal recovery is the metal removal efficiency (*η_Au_* in%) determined using Equation (2), where *C_0_* and *C_t_* are the initial and the final gold concentrations (mM), respectively. For literature comparison, metal removal capacity (*q* in g_Au_ g_cell_^−1^) was determined from Equation (3), where *V* is the volume of solutions in L, and *M_C_* is the mass of cells in grams.

(2) $$\mathrm{Removal}\;\mathrm{efficiency}\;(\eta_{Au})\;\eta_{Au}=\frac{C_{0}-C_{t}}{C_{0}}\times 100$$

(3) $$\mathrm{Removal}\;\mathrm{capacity}\;(q)\;q=\left(C_{0}-C_{t}\right)\times 10^{-3}\times \frac{V}{M_{c}}$$

Calculating *M_c_* is crucial for quantitative comparisons of metal ion recovery [32]. Here, it is used the allometric relationship between cell volume and weight applied previously for *S. putrefaciens* [32]. *M_c_* from Equation (4) considered the initial bacterial cell concentration (*C_b_*) in the reactors (cells mL^−1^), *V_c_* is the volume of the cells injected into the reactors in mL (typically 5 or 10 mL), and *M_b_* is the bacterial mass (g cell^−1^). *M_b_* was calculated from Equation (5), where *V_b_* is the cell volume in µm^3^, considering a conversion factor between weight/volume and a scaling factor. The volume of bacterial cells, Equation (6), was calculated considering the width (*w*) and length (*l*) of 25 bacterial cells unexposed to metal ions from TEM analysis.

(4) $$\mathrm{Mass}\;\mathrm{of}\;\mathrm{cells}\;\mathrm{applied}\;M_{c}=C_{b}\times V_{c}\times M_{b}$$

(5) $$\mathrm{Bacterial}\;\mathrm{cell}\;\mathrm{weight}\;M_{b}=\left(435V_{b}^{0.86}\right)\times 10^{-15}$$

(6) $$\mathrm{Bacterial}\;\mathrm{volume}\;V_{b}=\left[\left(w^{2}\times \frac{\pi }{4}\right)\left(l-w\right)+\left(\pi \times \frac{w^{3}}{6}\right)\right]$$

Bacterial dimensions of *S. oneidensis* MR-1 (*w* = 0.62 ± 0.08 and *l* = 1.34 ± 0.64 µm) and *C. metallidurans* CH34 (*w*= 0.55 ± 0.08 µm and *l* = 1.14 ± 0.35 µm) were comparable to a previous characterization of *S. algae* [22] and *C. metallidurans* [45]. Our average cell weight (*M_b_*) was 1.73 × 10^−13^ g cell^−1^ *S. oneidensis* MR-1 and 1.23 × 10^−13^ g cell^−1^ *C. metallidurans* CH34, which is comparable with the one for *S. putrefaciens* (1.28 × 10^−13^ g cell^−1^) reported by Varia et al. (2014).

While performing DOE analysis, Pareto plots indicate the standardized magnitude of effects for studied response variables, *η_Au_*, and average NPs_size_. The effect (*c_j_*) of each factor (*j*) is represented in Equation (7), where *m* is the number of reactors, and the response variable of each reactor is represented as observation. From these values, Lenth’s margin error (*ME*) (Equation (8)) evaluated which critical effects are greater than a random error [46]. Lenth’s is a non-parametric method applied for non-replica analysis in a saturated fractional factorial design. A pseudo-standard error (PSE) is derived from the median of the selected effects (c_j_*), after excluding the effects that exceed 2.5 times Lenth’s estimate (1.5 times the median of all effects, on the basis that null responses exceeding this value are negligible) [46]. Additionally, the significance level of effects was calculated, as indicated by a *p_value_* (95% confidence interval) using analysis of variance (ANOVA).

(7) $$Effect=c_{j}=\left|\frac{2}{m}\times \sum_{i=1}^{m}\left(algebraic\;sign_{\left(1,-1\right)}\times observation_{i}\right)\right|$$

(8) $$ME=2.297\times PSE=2.297\times \left(1.5\times (\tilde{x}\;of\;c_{j}^{*})\right)\;;\;c_{j}^{*}=\left|c_{j}\right|<3.75*\tilde{x}\;of\;c_{i}$$

## 3. Results and Discussion

### 3.1. Valuable Gold Nanoparticles and Gold Recovery Are Tailored by pH and Initial Metal Concentration

**Figure 1:**
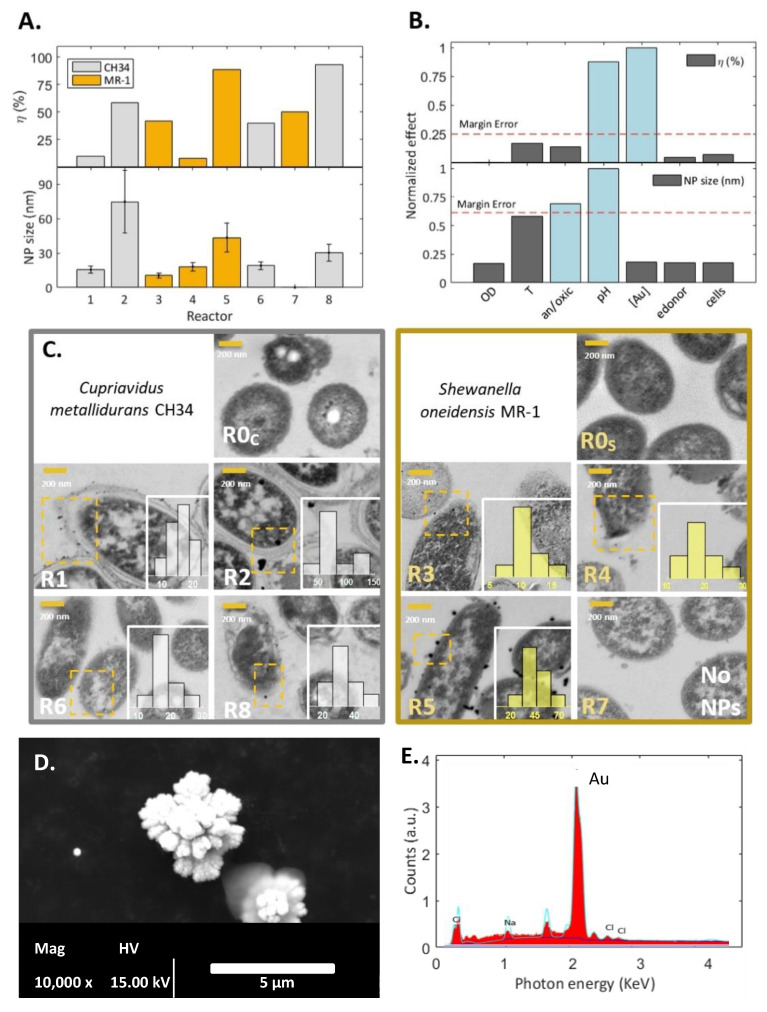
Fractional factorial (DOE) results for Au recovery and nanoparticle synthesis, (**A**) gold removal efficiency (η_Au_) and NPs size (NP_size_) in the 8 reactors with conditions from an orthogonal array of 7 factors (cell concentration, temperature, anoxic/oxic conditions, pH, initial gold concentration, electron donor type, bacterial species). (**B**) Pareto chart of the effect of each factor for response variable η_Au_ and NP_size_. Effect values are normalized from 0 to 1, by considering the effects quotient of the most significant effect. Significant effects lie out of the margin error. (**C**) TEM analysis and NPs size distribution (nm) for the 8 reactors (R1-8) and bacterial controls without metals (R0c and R0s) for C. metallidurans CH34 (gray) and S. oneidensis MR-1 (yellow). R7 (**D**) SEM characterization of AuNPs in suspension and (**E**) EDX.

Results for Au removal efficiency (*ƞ_Au_*) and nanoparticle synthesis based on the fractional factorial design of experiments (DOE, RES III) after three days of incubation are presented in Figure 1. The incubation time was selected as the removal rate typically approached zero after that period. 

Reactors R2, R5, and R8 provided the highest removal efficiency (Figure 1A), having in common the low concentration of Au^3+^ (i.e., 0.2 mM). These three reactors displayed a color change in the solution from pale yellow to vivid purple (Figure S1), which is typical of the formation of AuNPs [43]. This coloration is especially characteristic of large NPs (size > 30 nm) due to their interaction with light [43], which is a phenomenon known as surface plasmon resonance (SPR). No further color changes were observed after 3 days. 

The most significant factors to maximize *ƞ_Au_* were the initial metal concentration (*p_value_* = 0.001), and pH (*p_value_* = 0.002), according to ANOVA (*α* = 0.05). Pareto charts presented in Figure 1B corroborate this outcome, showing the significant effects (pH, [Au]) more pronounced than the margin of error (red line), which was calculated according to Lenth’s method [46].

A second analysis targeted AuNPs with a size of 50 nm, as catalytic properties [19,47] and potential application on surface-enhanced Raman scattering (SERS) are enhanced at that size [48]. In this fractional-factorial analysis, the significant factors according to ANOVA and Lenth’s method (Figure 1B) were pH (*p_value_* = 0.012) and oxic/anoxic conditions (*p_value_* = 0.029). 

An analysis of the factorial plot corroborated that a maximal gold removal efficiency can be obtained with 0.2 mM of initial [Au] and at pH 5 (Figure S2A). Similarly, AuNPs close to 50 nm in size (i.e., AuNPs_size_−50 approaches zero) are optimally obtained at pH 5 under anoxic conditions (Figure S2A).

The removal capacity related to the bacteria cells (*q*) was obtained for the eight reactors (Figure S3), showing the highest reported removal capacity for *Shewanella oneidensis* species in the conditions of reactor 7 (*q* = 28 g_Au_ g_cell_^−1^ ). Other studies only obtained 0.1 g_Au_ g_dry biomass_^−1^ [49] and 0.05 g_Au_ g_dry biomass_^−1^ with different bacterial strains and environmental conditions (Table S1). The influence of the ratio of the number of cells exposed to the metal ions in solutions was discarded, as higher initial cell concentrations (i.e., R4 and R6) did not show a higher *η_Au_* (Figure 1B) nor removal capacity *q* (Figure S3) than those with lower initial cell concentration (R1 and R7). 

High metal concentration levels in a solution can affect removal capacity by increasing toxicity towards the cells. Bacteria may develop resistance as a defense mechanism towards toxicity, but this regulation is often observed below the minimum inhibitory concentration (MIC) [30,50]. Concentrations of AuCl_4_^−^ above the MIC, i.e., here at 2 mM, can be considered highly toxic for the cells and thus decrease the gold recovery yield [24,50]. Therefore, lower *ƞ_Au_* with initially viable cells at a concentration of 2 mM AuCl_4_^−^ concentration can be explained due to increasing metal toxicity. Metal toxicity toward cells can also affect cell wall permeability [28], which would explain the presence of intracellular and extracellular NPs (see Figure 1C).

The protonation of the bacterial cell wall is influenced by pH, which suggests that a higher passive electrostatic adsorption of the AuCl_4_^−^ anions may occur at acidic pH [32,51]. However, our results showed a higher *η_Au_* at pH 5 rather than pH 1 (Figure 1A). Other authors have corroborated this finding, wherein the surfaces of the *S. oneidensis* MR-1 [17] and *S. haliotis* [52] are in equilibrium between positively and negatively charged groups with enhanced AuNPs synthesis at pH 5 [17,52]. The explanation relies on the most representative functional groups responsible for metallic biosorption in the cell wall of *S. oneidensis* MR-1, which are the amide, phosphoryl, and carboxyl groups. The amide groups (p*K*_a_ = 9.5–10.3) show a less noticeable interaction, as they are embedded on the cell wall deprotonated at the tested pH 5 [53]. Phosphoryl groups (p*K*_a_ = 6.8) maintain most of their positive charge at pH 5, contributing to AuCl_4_^−^ anion attraction. The carboxylic groups (p*K*_a_ = 4.7–6) dominate the outermost bacterial surface, so that at pH 5, a constant surface potential arises [53], indicating no alteration in electrostatic attraction (e.g., negative and positive ions are equally attracted to the charged groups of the cell membrane). The last may promote nucleation sites and colloidal stability that are less noticeable when the pH moves closer to neutral [17]. 

Although the selection between the type of an electron donor was not significant for *η_Au_* (Supplementary Data), an H_2_ headspace was present in all configurations where reactors exceeded *η_Au_*, >80%, and produced valuable AuNPs ≈ 50 nm. Konishi et al. reported the AuNPs recovery of up to 1 mM AuCl_4_^−^ (339.79 mg L^−1^) to be H_2_ dependent [54] by the involvement of common periplasmic hydrogenase for the *Shewanella* genus [9]. Although hydrogenase can play a significant role in gold reduction, De Corte et al. claimed that this periplasmic enzyme did not participate in gold recovery for *S. oneidensis* MR-1 [28]. Other co-occurring processes can be seen as crucial mechanisms, such as the physicochemical reduction of adsorbed metals on the cell wall and further metal nucleation. 

The TEM and SEM micrographs analysis showed that all experimental trials led to the precipitation of gold in different locations, morphologies, and sizes, as illustrated in Figure 1C,D. 

Electron-dense particles observed in TEM micrographs are interpreted as AuNPs, which were not present before exposure to metal ions (R0 c,s in Figure 1C). Intracellular AuNPs in reactor 3 were slightly smaller (10.5 ± 2.1 nm) compared to those in the periplasmic space displayed in reactor 1 (15.6 ± 2.9 nm). Both AuNPs locations were displayed in reactor 4 (18.3 ± 3.7 nm) and 6 (19.1 ± 3.3 nm). Larger NPs were observed extracellularly or on the cell wall in reactors 2 (103 ± 43 nm), 5 (43 ± 13 nm), and 8 (31 ± 7 nm), whose solutions exhibited a visual SPR effect perceived as a purple-like color, as explained above. Likewise, these reactors also exhibited the highest level of gold removal: R8 (*ƞ_Au_* = 92.8%, *q* = 4.8 g gcell^−1^ Au^3+^) for *C. metallidurans* CH34 (*[C_0_]_low_*, pH 5, *[e^−^donor]_H2_*) and R5 (*ƞ_Au_* = 88.5%, *q* = 3.4 g gcell^−1^ Au^3+^) for *S. oneidensis* MR-1 (*[C_0_]_low_*, pH 5, *[e^−^donor]_H2_*) as the studied bacterial species. According to the fractional factorial analysis, the 50 nm-targeted size of NPs should be attained when anoxic conditions are provided, as in R5. 

After filtration, R5 and R8 retained a vivid purple color over the filter, and no NPs were determined from the filtrate solution by SEM-EDX. These colorless filtrates suggested that AuNPs were bound to the biomass or sequestered in an EPS matrix. Conversely, R2 and R6 displayed spherical particles in SEM micrographs (Supplementary Data), suggesting that *C. metallidurans* expressed extracellular reductive precipitation by active efflux mechanisms, principally at pH 5 [30]. However, AuNPs were found extracellularly at pH 1; thus, cell wall detachment of more oversized agglomerates can be proposed [22]. Although electron-dense particles surrounding the cell seem absent in the TEM micrograph of R7 (Figure 1C), gold micro-particles with a flower-like morphology were observed in suspension (Figure 1D). Few researchers have successfully synthesized 3D flower-like Au-structures using chemical reducing agents and stabilizers [55,56,57], but the mechanisms remain elusive. Here, a possible route can be proposed, (i) After reduction by a microbial reducing agent (46% removal Figure 1A), seeded gold Au^0^ nuclei could be formed extracellularly [14]. (ii) After removing the biomass (cells), the seeds in suspension start forming branched structures ending in this noteworthy morphology at the microscale. 

Desired AuNPs geometrical and synthesis location according to their application could be synthesized by tuning the appropriate significant parameters revealed here. The AuNPs size, morphology, and location are essential for further valorization. Targeted 50 nm AuNPs are optimal for catalytic and SERS applications [19,47,48], and here we showed the importance of an anoxic environment to achieve them. Cells binding to AuNPs can act as carriers for applications such as catalysis in removing pollutants or enhancing hydrogen production in microbial fuel cells [2,19]. The NPs in the inner cytoplasm would be inaccessible for direct application, but can be recovered by processes such as lysis, sonication [18], or biomass combustion [58]. Furthermore, extracellular NPs are claimed to be easier to recover [25], requiring only a filtration step. 

### 3.2. Viable Shewanella Oneidensis MR-1 Combined with Abiotic Metallic Reduction Achieves Higher Metal Recovery than Inactivated Cells or Abiotic Reactors

**Figure 2:**
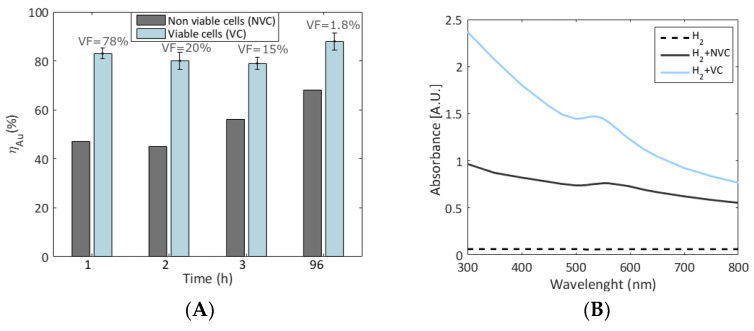
Comparison between heat-killed non-viable cells (NVC) and initially viable cells (VC) of *S. oneidensis* MR-1, following the conditions of R5 from DOE (0.2 mM of initial gold concentration, anoxic conditions, pH 5, and in the presence of H2 as an electron donor). (**A**) Gold removal efficiency (*η_Au_*) with time. VF values represent the percentage of remaining viable cells compared with the initial viable inoculated cells. (**B**) UV-Vis absorbance of an abiotic solution with hydrogen (H_2_), non-filtered solutions from treatment with initially viable cells and hydrogen (H_2_+VC), and biotic controls with heat-killed cells (H_2_+NCV). Further comparison is given in the Supplementary Data.

Based on the results mentioned above, the conditions of R5 with *S. oneidensis* produced functional AuNPs (≈50 nm) at a 0.2 mM initial gold concentration, under anoxic conditions, pH 5, and in the presence of H_2_ as an electron donor. Thus, validation experiments were carried out in triplicate with these conditions. Control experiments with autoclaved (heat-killed) cells and without bacteria were also conducted in the presence of H_2_. As illustrated in Figure 2A, *η_Au_* of the treatment at pH 5 was 88.2 ± 3.5%, comparable with the previous results from the DOE R5 (88.5%). Active and heat-killed cells were found to reduce a similar amount of H_2_ (≈44% from the headspace) during the experimental time. At the same conditions, H_2_ without the presence of bacteria could serve as an electron donor to reduce up to 65% of ionic gold (Au^0^ precipitates), whereas initially, heat-killed cells achieved up to 67.5% *η_Au_*. These results suggest that significant reduction can be attributed to physicochemical mechanisms, which is consistent with the report of De Corte [28].

UV-Vis spectra from the treatment with initially viable cells (VC) and hydrogen showed a clear peak at 540–550 nm (Figure 2B) as a quantitative indicator of higher AuNPs yield [9,43]. The control with initially heat-killed *S. oneidensis* MR-1 (NVC) showed a flattened peak at the same wavelength (Figure 2B), which corresponds with previous reports where putative “dead cells” produce fewer NPs in comparison with initially viable cells [28,32]. Due to cell lysis occurring by the heat treatment [28], AuNPs synthesis may result from the external exposure of reducing biomolecules (e.g., amino acids) [59] and other sorption sites. All these reports contradict the speculation that initially dead biomass can synthesize AuNPs more efficiently by disregarding the toxicity barrier of initially active cells [16]. Here, H_2_ alone did not induce AuNPs from abiotic AuCl_4_^−^ solutions (Figure 2B), which has also been reported in the literature [9,22]. Thus, bacterial cells were needed to catalyze the formation of valuable nano-precipitates.

Cell viability monitored by FC analysis indicated that at pH 5, the bacterial cells remained 78% intact for the first hour of the experiment (viability factor, VF, in Figure 2A), but >98% reflected membrane damage after 24 h. *Shewanella* sp. can thrive at pH 5 in a rich medium [60]; thus, the effect could be attributed to the AuNPs formation interfering with the cell wall. The pH is critical for NPs formation, and it should be considered parallel to the Pourbaix diagram of the species present in the waste stream for efficient gold recovery. Initially, viable cells always had a higher *η*_Au_ than non-viable cells (Figure 2A). After 96 h (1.8% intact cells), the removal could be acquainted with passive mechanisms, such as agglomeration and nucleation of colloids. 

**Figure 3:**
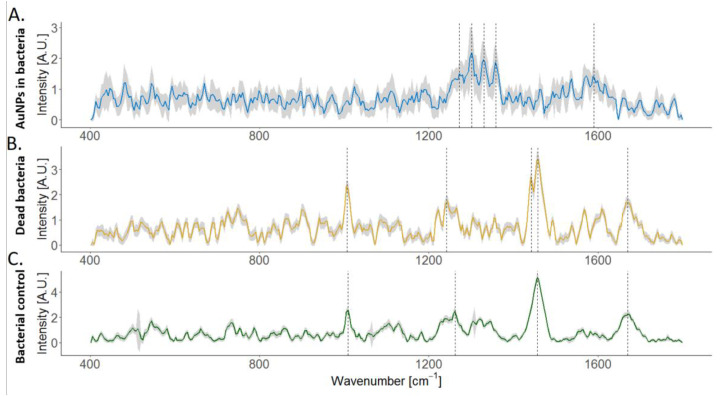
Raman spectra based on R5 conditions (0.2 mM of initial gold concentration, anoxic conditions, pH 5, and in the presence of H_2_ as an electron donor), (**A**) initially viable *S. oneidensis* that produced AuNPs (number of cells considered n = 128); (**B**) heat-killed *S. oneidensis* that produced fewer AuNPs (n = 44) (**C**); *S. oneidensis* without exposure to the metal as control (n = 114). The standard deviation is shown in gray.

As shown in Figure 3, some Raman spectral regions are enhanced with the presence of AuNPs. The results of the initially viable cells forming AuNPs showed Raman enhancement at regions corresponding to cytochrome-c, which is involved in active microbial mechanisms of gold reduction, and others as lipids, nucleic acids, and DNA (Figure 3A, Table S4). Some of the most intense peaks found in this study had already been reported in Wu et al. (2013), i.e., nucleic acid peaks at 1318, 1347, and 1580 cm^−1^ [61]. The heat-killed bacteria that produced fewer AuNPs showed different intense Raman regions than the initially viable bacteria producing AuNPs. For instance, cytochrome-c is not among the most intense spectra; other regions that correspond to nucleic acids, proteins, or lipids show an intense band (Figure 3B, Table S5). The bands observed are aligned with reported microbial-mediated AuNPs synthesis involving capping agents (lipids or proteins) [25], which act as nucleation sites and prevent their agglomeration. Bacterial control, without exposure to metal ions, resembles the spectra of heat-killed bacteria. Thus, the denaturation of protein by heat-killed cells that could influence the lower removal of gold [62] was not detected by Raman. These results align with the findings of Wu et al. (2013), where they found cytochromes relevant for AuNPs formation, but not essential. Further investigation is suggested to validate this result.

### 3.3. Provision of In-Situ Electron Donor for Higher Metal Recovery by Electrochemical Systems

A cathodic compartment of an electrochemical system can provide hydrogen by water reduction during the electrolysis of aqueous matrixes. In this research, a preliminary study was performed by introducing the selected microbial precursor, *Shewanella oneidensis*, in the cathodic compartment of an electrochemical system (set-up described in Figure S7). This configuration obtained a higher gold removal of 97.1% after 24 h of electrolysis when the cathode was poised at −0.3 V vs. Ag/AgCl, at a presumptive production of H_2_ (Figure S8). An abiotic control under the same conditions without the bacteria electrochemically removed only 57% of gold. The latter was associated with an electroreduction of gold based on the cyclic voltammetry tests (Figure S8). Further reduction and removal of gold in the biotic electrochemical system occurred via ion adsorption, reduction, and nucleation in the cell. Thus, combining biological with electrochemical mechanisms transformed the gold from the aqueous solution into AuNPs when an in situ electron donor was provided.

**Figure 4:**
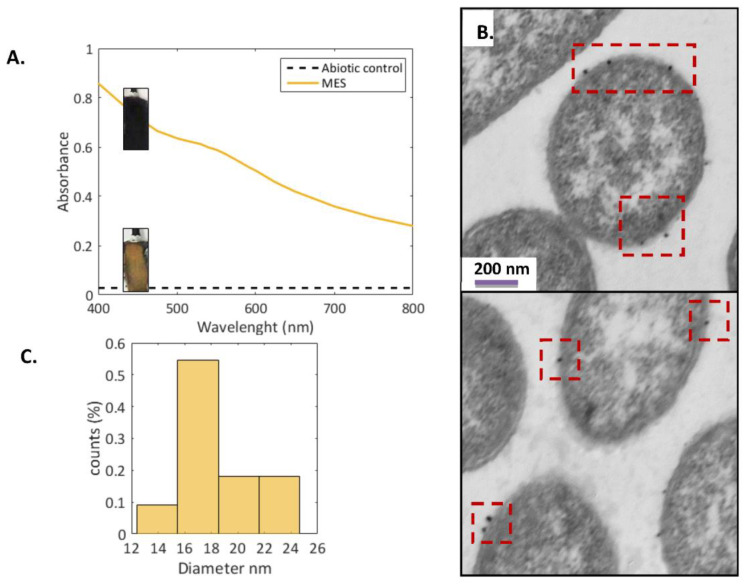
Confirmation of gold nanoparticle synthesis (AuNPs) in an electrochemical system with cathode potential fixed at −0.3 V vs. SHE. (**A**) UV-Vis absorbance of non-filtered catholyte of BES compared with the abiotic control; (**B**) TEM of S. oneidensis MR-1 after 72 h of operation; (**C**) AuNPs size distribution from TEM micrograph.

The UV-Vis spectra of the aqueous solution in the cathodic compartment exhibited a peak at 550 nm when the bacteria was introduced, indicating AuNPs formation [9,43] (Figure 4A). Conversely, the absence of AuNPs in the abiotic electrochemical system is explained by the lack of capping agents that promote the colloidal stability for gold nanoparticles [16,25]. Surprisingly, no gold deposits were observed on the cathode in the presence of bacteria (Figure 4A, see insets of carbon electrodes). Rapid sorption of gold on the bacterial cells and sorption of bacteria on the cathode could prevent the formation of golden layers on the cathode surface. Thus, the reduction and precipitation of AuNPs would likely occur in the surroundings of the bacteria (Figure 4B). The AuNPs attached to the bacteria in the electrochemical strategy had an average size of 18 ± 2.7 nm (Figure 4C). They were smaller compared to non-electrochemical mediated biosynthesis (Figure 1C). Suggested reasons for these variations presumably include (i) faster kinetics that could induce smaller AuNPs [63], (ii) a possible limitation of the in situ electron donor provision (e.g., whether hydrogen was produced) could influence the smaller AuNPs size, as with an excess of electron donors larger NPs have been observed [9]. 

To note, this manuscript has described the synthesis of AuNPs by two bacterial strains (*S. oneidensis* and *C. metallidurans*) with a discussion supported by the most relevant literature related to the current scope. Further data comparison of the recovery capacity for other metallic NPs synthesis [64] or employing other *Shewanella* strain [65] are available in Supplementary information. One review of biosynthesis of metallic nanoparticles [66] served as a basis for redrafting an overall table of the current studied factors and response variables for diverse bacteria strains [67,68,69,70,71,72,73,74,75,76,77,78,79,80,81,82,83,84,85].

## 4. Conclusions

The solely microbial alternative can reduce most of the gold concentration in solution (88.2 ± 3.5%) and produce valuable nanoparticles. Factors such as pH and initial metal concentration have a higher effect in the removal of metal ions in solution, disregardless of the type of bacteria cell, cell concentration, and viability of the bacteria cell. Despite respiratory strategies of the *Shewanella oneidensis* being able to enhance Au removal, biosorption is presented as the main mechanism for removal and AuNPs production. A more sustainable provision of electron donors was demonstrated by electrochemical systems, yet it demands further investigation to correctly estimate the production of the presumptive electron donor. Microbial strategies have the potential to provide an alternative solution for metal recycling from aqueous streams and concomitantly reducing cost and potential chemical wastes for the synthesis of metallic NPs in a green-chemistry approach. The use of costly reagents and postprocessing requirements to produce and stabilize gold nanoparticles can be replaced by microorganisms, thereby reducing the cost of synthesis operations.

## Figures and Tables

**Table 1:** Orthogonal matrix as experimental design for gold recovery applied in Resolution (III) fractional factorial of a design of experiments (DOE).

Experimental Order	Factor						
	OD_610_	Temperature	Anoxic/Oxic	pH	[Au^3+^] mM	e^−^ Donor	Bacterium
1 (R1)	0.5	37 °C	Anoxic	1	2	H_2_^b^	CH34
2 (R2)	1	28 °C	Anoxic	1	0.2	Lactate ^a^	CH34
3 (R3)	0.5	37 °C	Oxic	1	0.2	Lactate ^a^	MR-1
4 (R4)	1	28 °C	Oxic	1	2	H_2_^b^	MR-1
5 (R5)	1	37 °C	Anoxic	5	0.2	H_2_^b^	MR-1
6 (R6)	1	37 °C	Oxic	5	2	Lactate ^a^	CH34
7 (R7)	0.5	28 °C	Anoxic	5	2	Lactate ^a^	MR-1
8 (R8)	0.5	28 °C	Oxic	5	0.2	H_2_^b^	CH34

^a^ Lactate concentration = 10 mM. ^b^ H_2_ in saturation (≈0.9 mM at 25 °C).

## Data Availability

All data is included in this manuscript and the supplementary information.

## Supplementary Materials

The following supporting information can be downloaded at: https://www.mdpi.com/article/10.3390/nano13010083/s1, Figure S1. Photos of the 8 different conditions described in the orthogonal matrix for gold recovery with bacteria. At the beginning (upper picture) and after 48 h of incubation (bottom picture); Figure S2. Final Au^3+^ removal after 3 days of incubation. Factorial plots of relevant factors A: Au^3+^ removal efficiency (ƞ_Au_) and B: an optimal target for AuNPs size 50 nm; Figure S3. Removal capacity of bacteria cells applied. Shewanella oneidensis MR-1 and Cupriavidus metallidurans CH34; Figure S4. Filtrated solutions after 72 h of R2: i.A. SEM and i.B. EDX; and R6: ii. A. SEM and i.B. EDX; Figure S5. TEM micrographs after 72 h of (A) R7 at pH 2 and (B.i) R7 at pH 5. (B.ii.) SEM micrograph showing gold particles from the filtrated solution; Figure S6. Comparison between initially non-viable cells and intact cells. A. i. Initial flow cytometry panels of initially intact Shewanella oneidensis MR-1 on gold solutions, and ii. after 96h of treatment at conditions of Reactor 5. B.TEM micrographs for the conditions described; Figure S7. Diagram and description of the A: three recirculating reactors set up, where metal recovery experiments were conducted and B: two cylindrical stirring reactors, where standardized electrochemical analysis was derived consisting of a cylindrical cathodic compartment with a working volume of 150 mL, stirring at 350 RPM, with an inner concentric anode compartment of 20 mL separated with a Cation exchange membrane (CEM); Figure S8. Electrolyte analysis of the BES experiments. (A.) Cyclic Voltammetry (CV) of 0.2 mM Au^3+^ in 0.9% NaCl at pH = 5 before (t0) the potentiostatic test (chronoamperometry). I, II and III represent cathodic reaction regions. (B.) CV (scan rate = 20 mV s^−1^) from 0.2 mM Au in 0.9% NaCl pH = 2 a: without bacterial cells, b: with *S. oneidensis* MR-1 (≈0.5 × 10^8^ cells mL^−1^). Experiments performed with graphite electrodes and 28 °C in abiotic conditions as check-up tests (C) CV test after BES test showing higher hysteresis. (D) Electrolyte appearance after the potentiostatic test; the abiotic catholyte is clear whereas biotic catholyte reflects a purple coloration, related to the AuNP formation;Table S1. Current state of the art of reported in green synthesis of AuNPs via bacterial mechanisms; Table S2. Factors studied for gold recovery and the statistical analysis of the significant effect according to the design of experiments; Table S3. Raman specification during the analysis of gold validation results; Table S4. Most intense Raman peaks in initially active S. oneidensis with mediated AuNPs synthesis; Table S5. Most intense Raman peaks in heat-killed S. oneidensis with less AuNPs formation; Table S6. Most intense Raman peaks in S. oneidensis MR-1 control (without exposure to Au ions). The supporting information included various other reports for extended discussion [64,65,66,67,68,69,70,71,72,73,74,75,76,77,78,79,80,81,82,83,84,85].

## Abbreviations

Au, gold; AuNPs, gold nanoparticles; DOE, fractional factorials design of experiments; EPS, extracellular polymeric substances; MIC, microbial inhibitory concentration; e-donor, electron donor; OD610, optical density, FC, flow cytometry; SPR, surface plasmon resonance; TEM, transmission electron microscopy.
