# Supplementary material for: Combined Gold Recovery and Nanoparticle Synthesis in Microbial Systems Using Fractional Factorial Design
Source: Nanomaterials (Basel). 2022 Dec 24;13(1):83. doi: 10.3390/nano13010083 (PMC9824045; doi:10.3390/nano13010083)
Supplement: Supplementary file 1 [file nanomaterials-13-00083-s001.zip › nanomaterials-2102490-supplementary.pdf]

Combined gold recovery and nanoparticle synthesis in microbial systems using fractional factorial design

Supplementary Data

Table of Contents

Figures 2

Figure S1 2

Figure S2 2

Figure S3 3

Figure S4 3

Figure S5 4

Figure S6 5

Figure S7 7

Figure S8 8

Tables 10

Table S1. Factors studied for gold recovery and the statistical analysis of the significant effect according to the design of experiments 12

Table S2. Raman specification during the analysis of gold validation results 14

Table S3. Most intense Raman peaks in initially active *S. oneidensis* with mediated AuNPs synthesis 15

Table S4. Most intense Raman peaks in heat-killed *S. oneidensis* with less AuNPs formation .. 16

Table S5. Most intense Raman peaks in *S. oneidensis* MR-1 control (without exposure to Au ions) 17

Figures

Figure S1

At 48 h of experimentation, reactors 2, 5, and 8 showed a colour change from slightly yellow to a vivid purple colour (Figure S1). After that, no further colour change was observed.

R1 R2 R3 R4 R5 R6 R7 R8

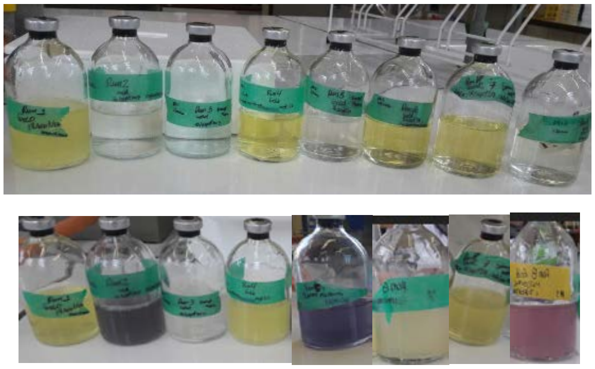

Figure S1. Photos of the 8 different conditions described in the orthogonal matrix for gold recovery with bacteria. At the beginning (upper picture) and after 48 h of incubation (bottom picture).

Figure S2

The factorial plots show that pH 5 at a low initial concentration of gold (0.2 mM Au^{3+}) provides the maximized response. On the other hand, as optimization of nanoparticles was set to 50 nm, the response variable $(AuNP_{size}-50)^2$ should look for outcomes closer to zero. With this in mind, anoxic conditions and pH 5 will promote this response (Figure S3 B)

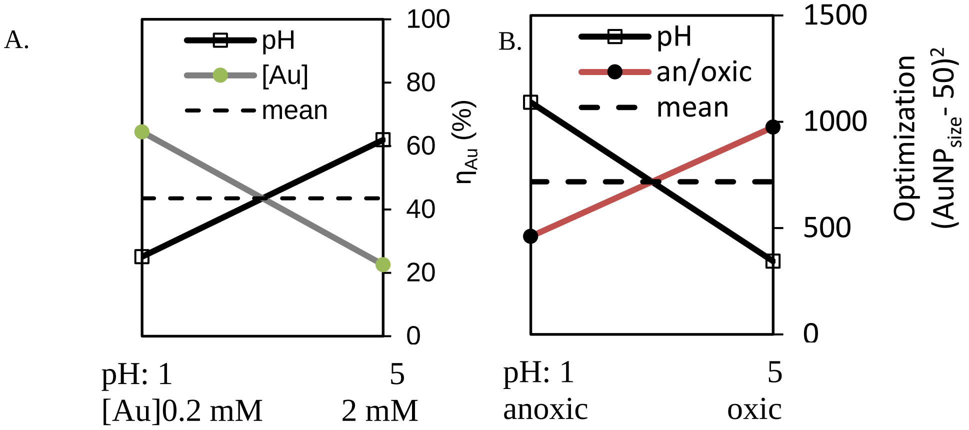

Figure S2. Final Au^{3+} removal after 3 days of incubation. Factorial plots of relevant factors A: Au^{3+} removal efficiency (η_{Au}) and B: an optimal target for AuNPs size 50 nm.

Figure S3

The affinity of *S. oneidensis* MR-1 is observed as higher gold removal capacity, especially under the optimized conditions at pH 5 (observed in R5 and R7). R7 achieved the highest metal removal capacity (q = 0.14 mol Au^{3+} g $cell^{-1}$) inoculated with *S. oneidensis* MR-1, which has a higher affinity than for chromium (0.015 mol Cr $gcell^{-1}$) [1]. Moreover, higher specific removal capacity (1.2 x 10^{-6} mol-Au $cell^{-1}$) with *S. oneidensis* is observed in comparison with *S. algae* cells (2.1 - 3.3 x 10^{-16} mol-Au $cell^{-1}$) [2].

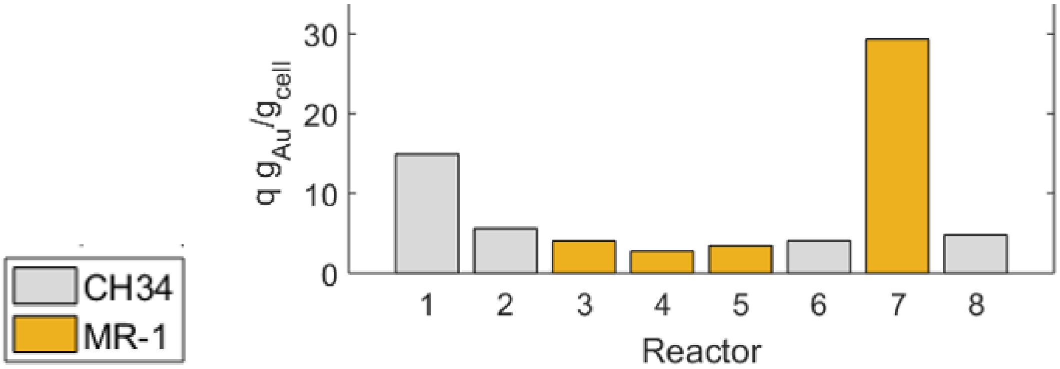

Figure S3. Removal capacity of bacteria cells applied. Shewanella oneidensis MR-1 and Cupriavidus metallidurans CH34

Figure S4

Filtrated solution (0.2 µm) for reactors 1, 3, 4, 5, and 8 did not reveal particles during SEM analysis, which is indicative that particles were mainly bound to the biomass. Figure S4 shows SEM micrographs of reactors 2 and 6, where spherical AuNPs were identified extracellularly.

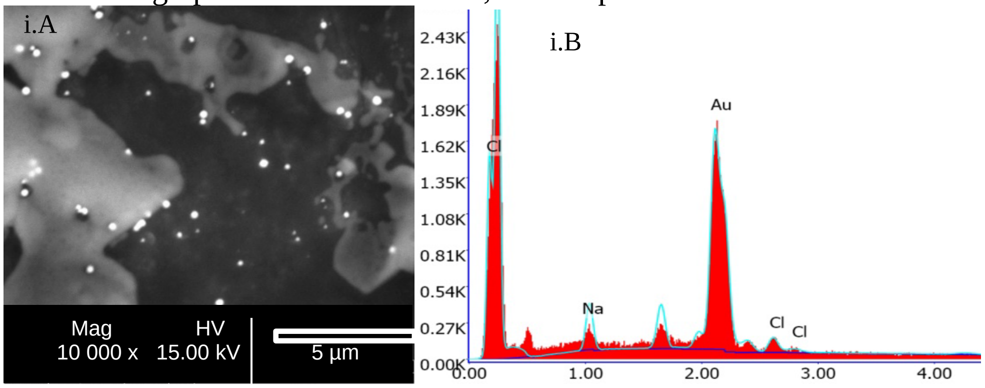

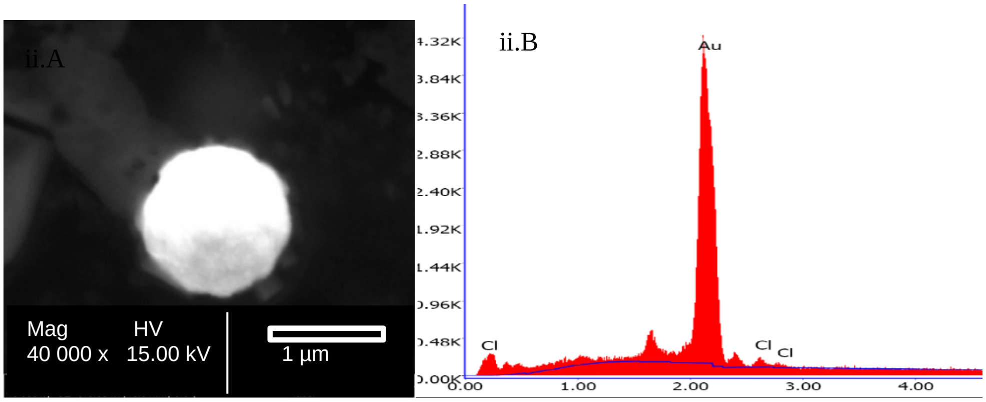

Figure S4. Filtrated solutions after 72 h of R2: i.A. SEM and i.B. EDX; and R6: ii. A. SEM and i.B. EDX.

Figure S5

TEM analysis was obtained from samples (1.5 mL) centrifuged (5000 relative centrifugal force, Eppendorf 5430) for 5 minutes at room temperature. The supernatant was discarded, and the pellet was fixed with 1 mL of 4% paraformaldehyde, 5% glutaraldehyde in 0.1 M cacodylate buffer (VWR, Merck, USA). Repeatedly, the samples were centrifuged and stained with OsO_4 (1% w/v dissolved in a 0.1 M Na-cacodylate buffer, VWR, Merck, USA). The sample was dehydrated stepwise with alcohol (50%, 70%, 90% absolute ethanol, VWR, USA). The pellets were then embedded at room temperature in Epon medium (EMS, EMBED 812 Resin, Netherlands). Semi-thin sections of 1 µm were first stained with toluidine blue, for initial visual observation of the cells. Subsequently, ultrathin sections (≈ 60 nm) were cut with a diamond knife and contrasted with uranyl acetate (94260 Fluka, Sigma-Aldrich, Canada) and lead citrate (7398, Merck, Germany).
R7 conditions at pH 5 were compared with similar conditions at lower pH 2. At pH 2, TEM revealed cell membrane disintegration. AuNPs agglomerate and flocculate in the material around the cell. Therefore, NPs are observed in the close vicinity of the cells and not extracellularly, as after filtration, they were not observed in SEM. Contrarily, at pH 5 TEM shows that the cell maintained their cell membrane integrity, but it is observed to expel cytoplasmic content. These expulsed biomolecules could be responsible for the nucleation of particles extracellularly, and as cell walls seem intact, the biomolecules released could be the main reducing agent. After forming the nuclei (Au^o), particles in the filtrated solution start to agglomerate in suspension as there is a higher binding force between them than the atom-solvent energy. Nonspecific interactions could lead to the microscale formation.

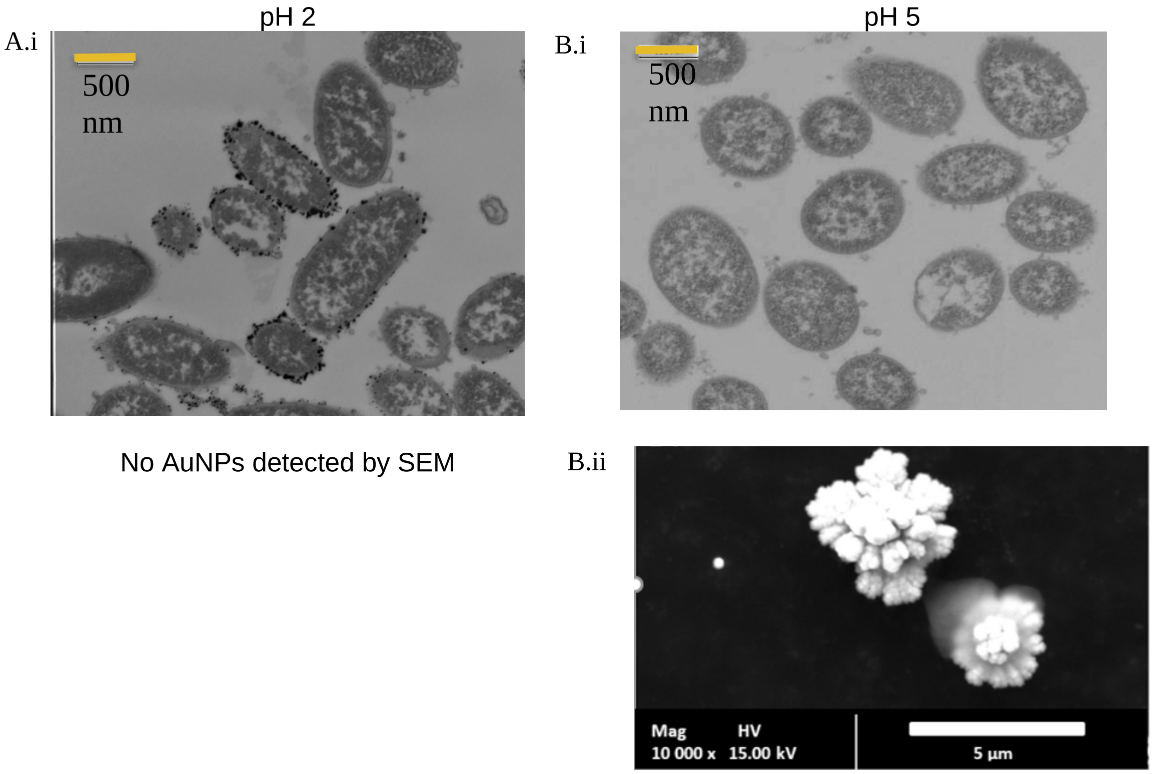

Figure S5. TEM micrographs after 72 h of (A) R7 at pH 2 and (B.i) R7 at pH 5. (B.ii.) SEM micrograph showing gold particles from the filtrated solution.

Figure S6

Flow cytometry panels are presented for comparison between initially active cells by at time 1h (Figure S6 Ai.) and after 96 h of gold exposure (Figure S6 Aii.). TEM shows that cell integrity is completely compromised when they are heat-treated (Figure S6 B i.). Smaller AuNPs tend to be scattered, and it is not possible to target the main active reducing site. However, in initially active cells, AuNPs agglomerate in specific sites around the cell wall

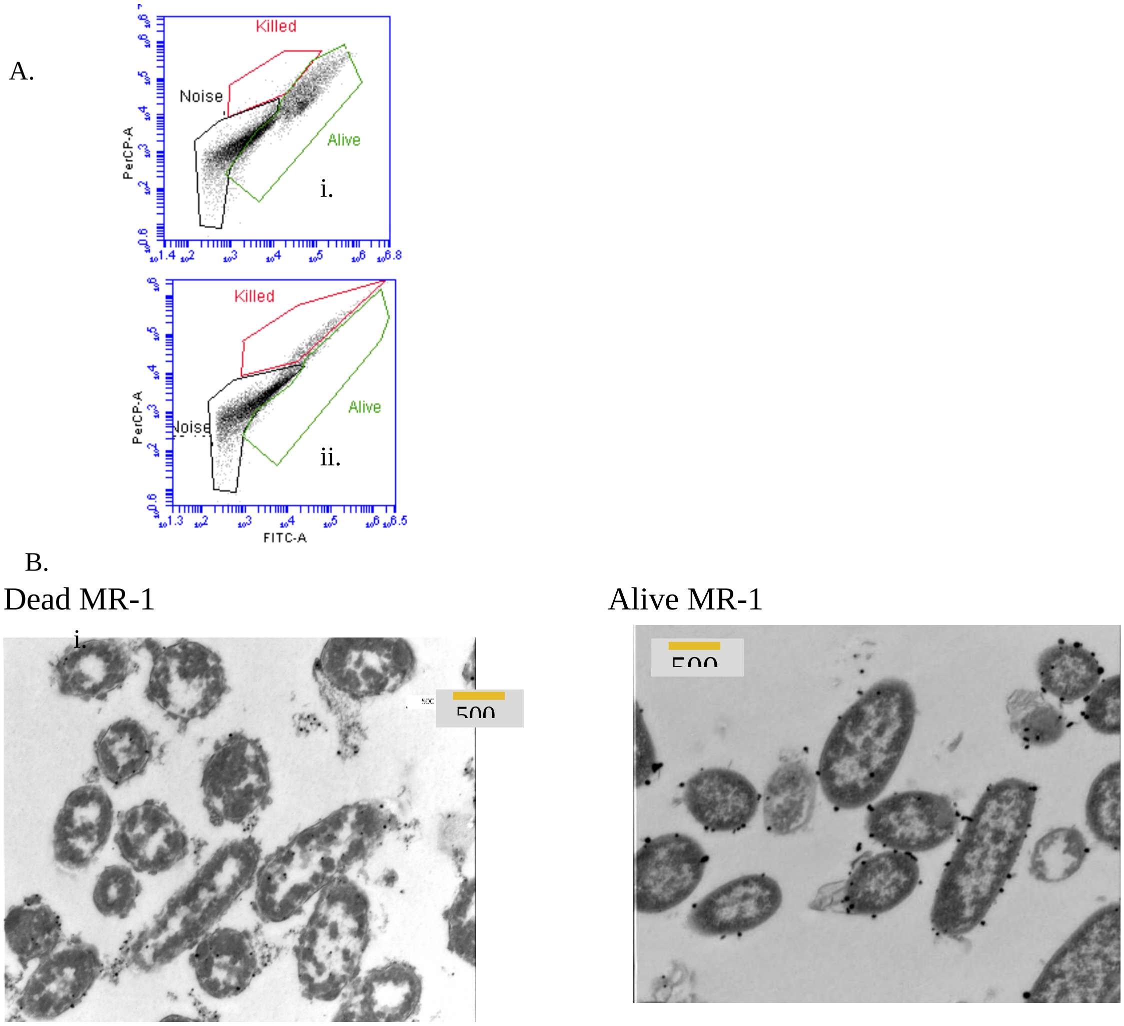

Figure S6. Comparison between initially non-viable cells and intact cells. A. i. Initial flow cytometry panels of initially intact Shewanella oneidensis MR-1 on gold solutions, and ii. after 96h of treatment at conditions of Reactor 5. B.TEM micrographs for the conditions described.

Figure S7

From the DOE, a validated condition that maximizes recovery was initially simulated in the cathodic chamber of an electrochemical reactor. Experiments were conducted in a temperature-controlled room (28 °C). Prior to tests, the recovery, electrochemical analysis for understanding the electrolyte nature and reaction evolutions on abiotic and biotic conditions were steered in a standard three-electrode system.

Microbial–electrochemical metal recovery was investigated in a two-chamber. The biotic system consisted of parallel Perspex frames with 0.13 L of working volume (with internal dimensions of 8 cm × 8 cm × 1.95 cm), separated by a cation exchange membrane (64 cm^2 CEM, Ultrex CMI-700, Membranes International, USA). A stainless-steel mesh (AISI 316L mesh size 495 µ, Solana, Belgium) functioned as an anode. The working electrode, cathode, was made of graphite plates (Mersem grade 1940 PT, France), with a projected surface area of 3 cm^2. An Ag/AgCl reference electrode (+ 0.2 V vs. SHE, 3 M KCl, Biologic Science Instruments, France) was placed closer to the cathode for electrochemical analysis and polarization during the experiment. All potentials were normalized against the standard hydrogen electrode (vs. SHE). The anolyte consisted of a 0.15 N Na2SO4, and the catholyte was the validated microbial test in a 0.9% NaCl, both adjusted at the same pH with 1 M NaOH. The catholyte was first purged with N_2 (Linde, Germany) for 15 minutes for tests under anoxic conditions. Bacterial cells were inoculated into the catholyte, as planktonic cells, at similar concentrations as in previous microbial tests without the provision of an additional carbon source. Recirculation of the electrolytes was set at 2.5 L h^{-1}, providing sufficient mixing in both compartments.

A potentiostat (Potentiostat/Galvanostat Model VSP, Biologic Science Instruments, France) controlled the system by applying a constant cathodic electron supply with presumptive H_2 evolution. Additionally, an abiotic control was conducted using identical reactor structure and electrolyte conditions without inoculating the microorganisms.

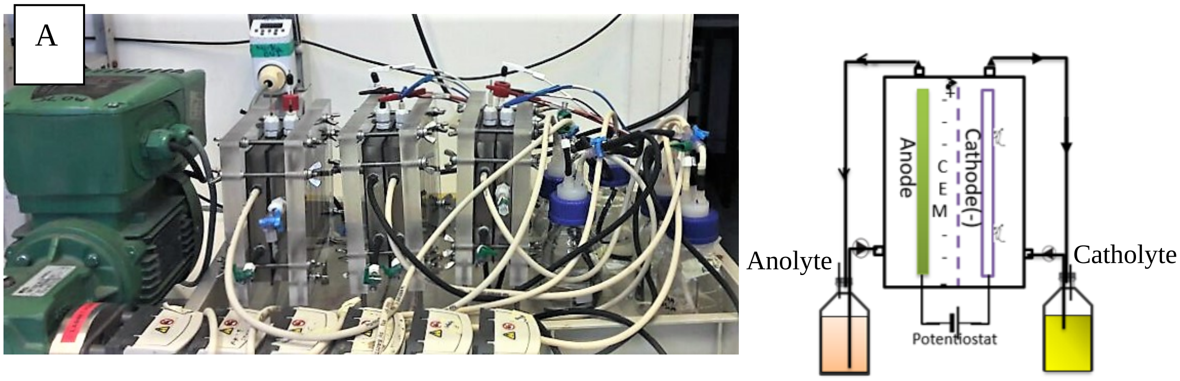

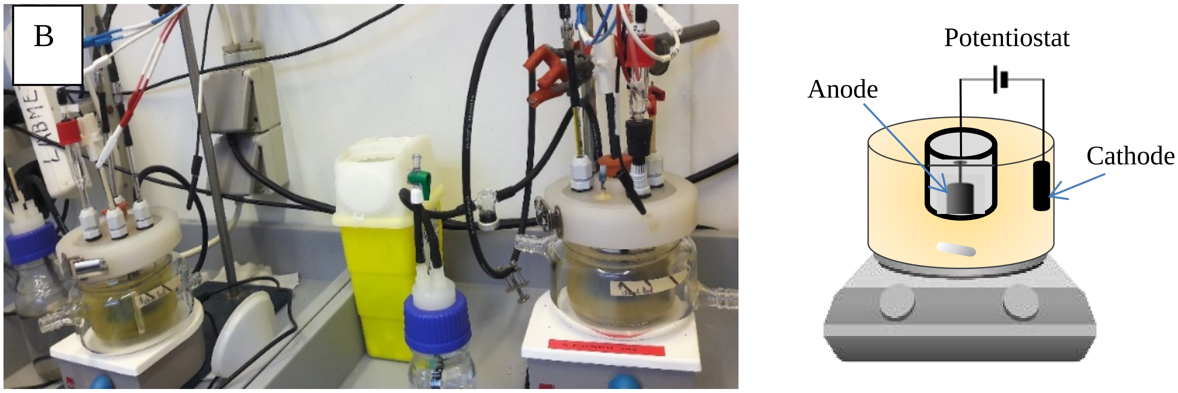

Figure S7. Diagram and description of the A: three recirculating reactors set up, where metal recovery experiments were conducted and B: two cylindrical stirring reactors, where standardized electrochemical analysis was derived consisting of a cylindrical cathodic compartment with a working volume of 150 mL, stirring at 350 RPM, with an inner concentric anode compartment of 20 mL separated with a Cation exchange membrane (CEM).

Figure S8

The electrochemical reactions and standard reduction potentials in the literature were used as a reference to interpret the cyclic voltammetry graph obtained in the experimental conditions.

$AuCl_4^- + 2e^- \rightarrow AuCl_2^- + 4Cl^-$ $\quad$ $E^0 = 0.95$ V *vs*. SHE

$AuCl_2^- + e^- \rightarrow Au^0 + Cl^-$ $\quad$ $E^0 = 1.15$ V *vs*. SHE

$AuCl_4^- + 3e^- \rightarrow Au^0 + 4Cl^-$ $\quad$ $E^0 = 0.8$ V *vs*. SHE

$2H+ + 2e- \rightarrow H_2$ $\quad$ E = -0.3 V vs. SHE

Cyclic voltammetry tests (CVs) at different scan rates showed three regions of interest (Figure S8A). The region around 1 V *vs*. SHE fits with the standard potential of $AuCl_4^-$ reduction to $AuCl_2^-$. Slower reduction from $AuCl_2^-/Au^0$ reaction can also be present. With a considerably higher current density response, a following cathodic region predominates between 0.5 and 0.9 V. This is most likely related to the 3-electron reaction of zero-valent gold electrodeposition and was assumed to be the main gold reduction reaction. The third region at negative cathode potentials includes H_2 generation and possible non-identified parasite reactions.

The electrochemical response of gold reduction with or without bacteria was also investigated. CVs show a more pronounced hysteresis in biotic conditions, suggesting a higher electrode capacitance. Bacteria in contact with polarized electrodes may act as considerably significant pseudo-capacitors. Furthermore, *S. oneidensis* can adsorb relatively fast on the polarized carbon surface, for instance, mediated by secreted redox species like flavines. The second region reaches a similar limiting steady-state current density followed by a faster depletion rate in biotic

conditions. Considering the rapid adsorption of metal ions in the bacteria cells, a gold diffusion limitation could be more pronounced in the presence of bacteria.

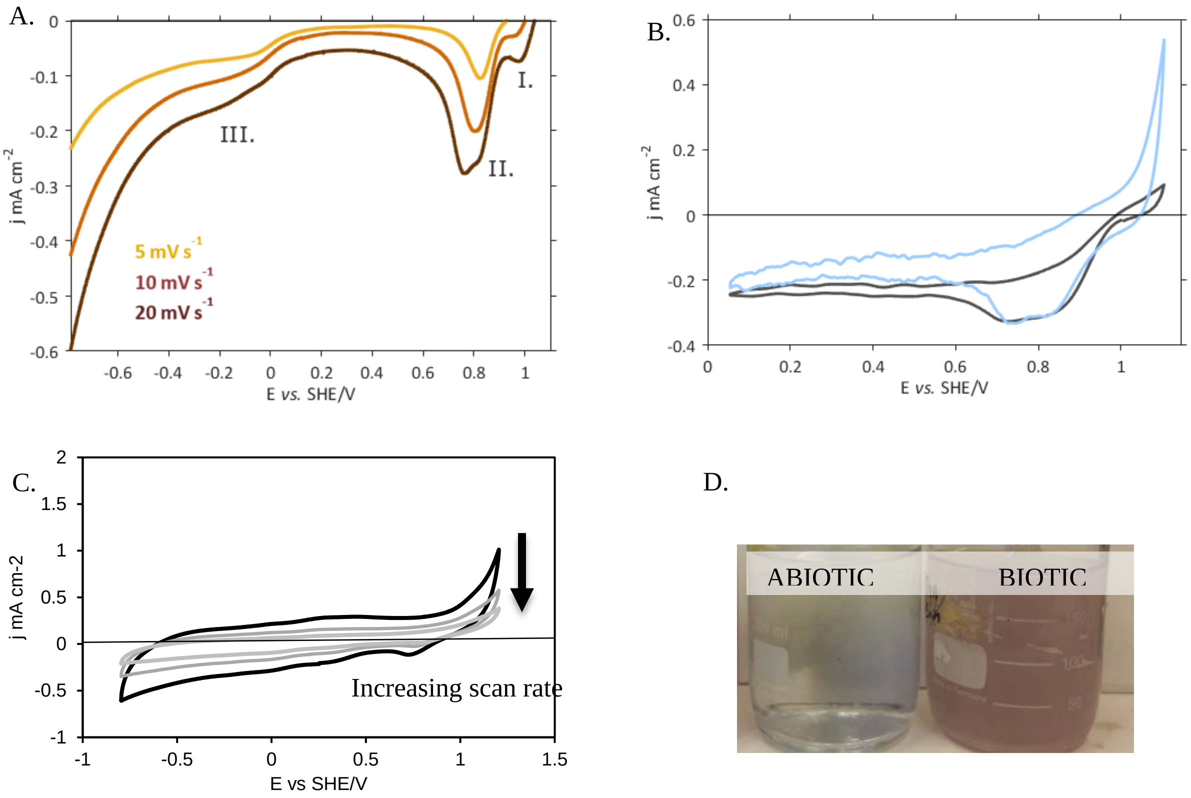

Figure S8. Electrolyte analysis of the BES experiments. (A.) Cyclic Voltammetry (CV) of 0.2 mM Au^{3+} in 0.9% NaCl at pH=5 before (t0) the potentiostatic test (chronoamperometry). I, II and III represent cathodic reaction regions. (B.) CV (scan rate = 20 mV s−1) from 0.2 mM Au in 0.9% NaCl pH = 2 a: without bacterial cells, b: with *S. oneidensis* MR-1 (≈ 0.5 x 108 cells mL-1). Experiments performed with graphite electrodes and 28 °C in abiotic conditions as check-up tests (C) CV test after BES test showing higher hysteresis. (D) Electrolyte appearance after the potentiostatic test; the abiotic catholyte is clear whereas biotic catholyte reflects a purple coloration, related to the AuNP formation.

Tables

Table S1. Current state of the art of reported in green synthesis of AuNPs via bacterial mechanisms. Redrafted from [3]

Microorganisms	Environmental conditions					Nanoparticles synthesis				Reference
	Aerobic / anaerobic	pH	Reducing agents	Gold concentration (mM)	Temperature (°C)	Size (nm)	Shape	Localization	Recovery capacity (Gold recovery %)	
Plectonema boryanum UTEX 485	Aerobic	8	-	2 - 2.8	60	–	Cubic	Membrane vesicles	(100%)	[4]
- Anabaena sp.<br>*- Calothrix sp.*<br>*- Leptolyngbya sp.*	Aerobic	≈ 7	-	0.001 - 1	25	6 - 12	–	Intracellular	-	[5]
Spirulina platensis	Aerobic	5,6	-	1	37	6–10	–	Extracellular	-	[6]
Lyngbya majuscula	Aerobic	6,7,8	-	0,05	25	<20 nm	Spherical	Intracellular and extracellular	0.002 g Au g^{-1} biomass (96%)	[7]
Sulfate reducing bacteria	Aerobic	≈ 7.4	Lactate	2,5	Room temperature	<10 nm	-	Cell envelope	-	[8]
Rhodobacter capsulatus	Aerobic	1 - 9	Lactate	0,25	30	–	–	Plasma membrane	0.054 mg Au/g dry weight of cell	[9]
Rhodopseudomonas capsulata	Aerobic	4 - 7	-	1	Room temperature	10–20	Spherical	Extracellular	-	[10]
Escherichia coli	Anaerobic	2, 6, 7, 9	H_2	2	37	5-50	Spherical	Periplasmic space, intracellular	(100%)	[11]
Escherichia coli DH5α	Aerobic	-	-	1	25	–	Spherical	Cell surface	-	[12]
Pseudomonas aeruginosa	Aerobic	-	-	1	37	15–30	–	Extracellular	-	[13]

Pseudomonas denitrificans	Aerobic	3		0,51	37	25–30	Face centered cubic	Cell bound		[14]
Shewanella algae	Anaerobic	7	H_2 - CO2, lactate	2,4	30	10–20	–	Periplasmic space, bacterial envelope	(100%)	[15]
Shewanella oneidensis	Aerobic	-	-	1	30	12±5	Spheres	–	-	[16]
Marinobacter pelagius	Aerobic	5-6	-	1,3	37	10	Triangle, spherical, polygonal	Cell wall bound	-	[17]
Bacillus megaterium DO1	Aerobic	3,2	-	0,5	26	1.9 ± 0.8	Spherical	Extracellular	0.12 g Au/ g dry biomass (99%)	[18]
Geobacillus stearothermophilus	Aerobic	6-7	-	1	27	12	Polydispersed, circular	Extracellular	-	[19]
Jeotgalibacillus sp.	Aerobic	3	Sodium citrate	1	30	5–35	-	Intracellular	-	[20]
Brevibacterium casei	Aerobic	-	-	1	37	10–50	–	–	-	[21]
Thermomonospora sp.	Aerobic	9	-	1	50	7-12	Spherical	Extracellular	-	[22]
Rhodococcus sp.	Aerobic	≈ 7	-	1	27	5–15	–	Intracellular	-	[23]
- *Arthrobacter sp.*<br>- *Arthrobacter globiformis 151B*	Aerobic	-	-	1	Room temperature	8–40	Spherical	Cell wall	-	[24]
Desulfovibrio desulfuricans	Anaerobic	7	H_2	2	30	5-50	Spherical	Periplasmic space, intracellular	(100%)	[25]

Table S2. Factors studied for gold recovery and the statistical analysis of the significant effect according to the design of experiments

Factor	Level (-1, 1)	Support of selected levels	References
pH	1 , 5	Tetrachloraurate ($AuCl4^-$) is predominantly present in solutions at pH < 6; thus, $AuCl4^-$ reduction reactions to metallic Au0 can occur in acidic electrolytes close to neutral pH.<br>Gold biosorption and bioprecipitation have been reported to be effective at pH 1 and 5	[26–28]
[Au]<br>Initial gold concentration	0.2 , 2 mM	God concentration in waste streams ranged from 0.005 - 10 mM<br>Bio recovery of gold via nanoparticles formation by bacteria has been reported from 0.2, 0.9 mM, 1mM, 2mM	[2,25,30,31]
e^- donor	H_2, Lactate	The studies of gold recovery *via* Nanoparticle formation involved an electron donor: typically lactate or hydrogen gas	[2,25,30,31]
Bacterial species	S. oneidensis MR1, Cupriavidus metallidurans CH34	Their respiratory strategies and its value demonstrate *Shewanella species* to be nanofactories.<br>*Cupriavidus* were able to produce gold nanoparticles *via* a defense mechanism	[2,30,32]

Cell biomass<br>Optical density	0.5 , 1	An optical density of 1, corresponding to a cell density of ¯10^7 cells/mL, which was within typical cell concentrations for bio recovery	[30,31]
Temperature and headspace	28, 37 °C<br>N_2, air	Influence microbial activity/metabolisms. promoting anaerobic respiration	[30,31]

Model	Factors in the model	Effect	Coefficient	T-Value	P-Value
$\% Au^{3+} removal$	Constant		48.74	21.27	0.00
η_{Au}	pH	38.20	19.10	8.34	0.002
	[Au]	-43.63	-21.82	-9.52	0.001
$AuNPs\ target$	Constant		685.5	9.30	0.003
	Temperature	497.6	248.8	3.37	0.043
	An/oxic	578.8	289.4	3.93	0.029
	pH	-812.0	-406	-5.51	0.012

Significant factors were determined by ANOVA and Lenth's method for 2 response variables: gold removal efficiency and AuNPs targeted size. Table S1 shows the summary of the statistical coefficients. Temperature at 37 ^{0}C would likely influence the AuNPs synthesis at the targeted size, revealed as significant by ANOVA (p_{value}=0.043) but only close to the marginal error by Lenth's un-replicated analysis. Anoxic conditions promote the respiration (e- acceptor) of the metals, while higher temperatures could increase metabolic reduction reactions, thus establishing nucleation sites for the AuNP formation.

Table S3. Raman specification during the analysis of gold validation results

Samples and sample acquisition	
Material and source	Shewanella oneidensis MR-1 LMG 19005
Growing conditions/sampling	Cells were grown at 28°C, 120 rpm
Label in the samples	No label used
Fixation method	Filtered PFA 4%
Integration time	40 second
Accumulations	1
Grid	300 –mm/g
**Instrument**	
Laser power	785 nm excitation diode laser (Toptica)
Silica check (quality control)	Before objective/after objective or just the total laser power: stability regarding other days was checked
Objective used (magnification) / Numeric aperture (NA)	100x/0.9 NA (Nikon)
Camera	-70 °C cooled CCD camera (iDus 401 BR-DD, ANDOR)
Dry/water/oil objective	Dried samples
Model of spectroscope	WITec Alpha300R+
Other specifications (chromatic/flat field correction/other)	
**Data analysis**	
Background subtraction method (if used)	No. Repeated measurements with cosmic rays
Normalization method (peak /min-max /area under-curve /other)	Area under the curve ('Total Ion Count')
Smoothing and interpolation (if done)	Baseline correction
Statistics/Machine learning algorithm	'MicroRaman' package (GitHub). Spectral contrast angle, ward.D2 dissimilarity and hierarchical clustering. Random forest
Accessibility	Repository: 'MicroRaman', GitHub
Other relevant information	Peak selection 900-1100 cm^{-1} removed for unexplained variation

Non-treated bacteria were measured with a 10 second time exposure and 1 accumulation. Microbial treatments leading to AuNPs synthesis were measured with a 0.5-second exposure time and 1 accumulation. The Raman spectra were cut in the 400-1800 cm^{-1} region, went under baseline correction (10 iterations) and normalization following the steps from the R package "MicroRaman" [1]. Peaks that presented a cosmic ray were manually removed.

[1]. Kerckhof, F. M.; Buysschaert, B.; Khalenkow, D.; Garcia-Timermans, C. MicroRaman. Github: https://github.com/CMET-UGent/MicroRaman 2018.

Table S4. Most intense Raman peaks in initially active *S. oneidensis* with mediated AuNPs synthesis

Wavelength (cm-1)	Mean intensity (A.U.)	Tentative peak assignment (closest identified peak, cm-1)
**1272**	1.50	*δ* (C=CH) (1273)
**1276**	1.42	Amide III (*α*-helix) (1279)
**1280**	1.46	Amide III (*α*-helix) (1279)
**1290**	1.48	Cytosine (1287-1290)
**1294**	1.58	CH_2 deformation (1295)
**1297**	1.99	CH_2 deformation (1295)
**1301**	2.18	Amide III (protein) (1302)
**1305**	1.99	CH_2 deformation (lipid), adenine, cytosine (1304)
**1308**	1.43	cytochrome c (1311)
**1312**	1.48	cytochrome c (1311)
**1326**	1.84	Phospholipids; DNA (1330)
**1329**	1.95	Phospholipids; DNA (1330)
**1333**	1.89	CH_3,CH_2 wagging mode of collagen and polynucleotide chain (DNA purine bases) (1335)
**1336**	1.53	CH_3,CH_2 wagging mode of collagen and polynucleotide chain (DNA purine bases) (1335)
**1354**	1.56	Guanine (N_7, B, Z-marker) (1355)
**1358**	1.87	Guanine (N_7, B, Z-marker) (1357)
**1361**	1.74	Guanine (N_7, B, Z-marker) (1361)

**1365**	1.44	Guanine (N_7, B, Z-marker) (1365)
**1572**	1.43	C=C, N-H deformation; C-N stretching (amide II) (1573)
**1588**	1.40	Cytochrome C (1583)

Table S5. Most intense Raman peaks in heat-killed *S. oneidensis* with less AuNPs formation

**Wavelength (cm-1)**	**Mean intensity (A.U.)**	**Tentative peak assignment (closest identified peak, cm-1)**
**1005**	2.34	-
**1009**	2.25	-
**1012**	1.87	-
**1240**	1.65	Asymmetric phosphate [$PO2^-$ (asymmetric)] stretching modes (1240)
**1244**	1.75	Guanine, cytosine (NH2) (1247)
**1438**	2.29	CH2 and CH3 deformation vibrations (lipid) (1440)
**1441**	2.7	CH2 and CH3 deformation vibrations (lipid) (1440)
**1445**	2.33	CH2 bending mode of proteins and lipids (1446)
**1448**	2.11	CH2 bending (1446)
**1452**	2.78	CH2/CH3 deformation, PHB (1451-1453)
**1455**	3.37	Overlapping asymmetric CH3 bending and CH2 scissoring (elastin, collagen, and phospholipids) (1454)
**1459**	3.41	Nucleic acid modes (1458)
**1462**	3.05	Fermi interaction δ (CH2) and γ (CH2) (1463)
**1465**	2.52	Lipids (1465)

**1469**	2.03	Lipids (1465)
**1472**	1.67	-
**1665**	1.69	-
**1669**	1.69	Protein band; C=C stretching band (1670)
**1672**	1.69	Ceramide (1672)
**1675**	1.63	Ceramide (1676)

Table S6. Most intense Raman peaks in *S. oneidensis* MR-1 control (without exposure to Au ions)

**Wavelength (cm-1)**	**Mean intensity (A.U.)**	**Tentative peak assignment (closest identified peak, cm-1)**
**1005**	2.39	-
**1009**	2.55	-
**1258**	2.24	Amide III, adenine, cytosine (1258)
**1262**	2.46	Thymine, Adenine; =C-H bend (protein) (1263)
**1438**	2.20	CH_2 and CH_3 deformation vibrations (lipid) (1440)
**1441**	2.64	CH_2 and CH_3 deformation vibrations (lipid) (1440)
**1445**	3.11	CH_2 bending mode of proteins and lipids (1446)
**1448**	3.85	CH_2 bending mode of proteins and lipids (1446)
**1452**	4.57	CH_2 bending (1450)
**1455**	5.04	Overlapping asymmetric CH_3 bending and CH_2 scissoring (elastin, collagen, and phospholipids) (1454)
**1459**	5.06	Nucleic acid modes (1458)
**1462**	4.56	Fermi interaction $\delta(CH_2)$ and γ (CH_2) (1463)
**1466**	4.00	Lipids (1465)
**1469**	3.28	Lipids (1465)
**1473**	2.70	-
**1476**	2.19	-
**1665**	2.15	-

**1669**	2.20	C=C stretching vibrations of cholesterol; Amide I (1670)
**1672**	2.25	Ceramide (1672)
**1675**	2.15	Amide I (β-sheet) (1676)

References:

[1] S.S. Middleton, R.B. Latmani, M.R. Mackey, M.H. Ellisman, B.M. Tebo, C.S. Criddle, Cometabolism of Cr(VI) by Shewanella oneidensis MR-1 produces cell-associated reduced chromium and inhibits growth, Biotechnol. Bioeng. 83 (2003) 627–637. https://doi.org/10.1002/bit.10725.

[2] Y. Konishi, T. Tsukiyama, K. Ohno, N. Saitoh, T. Nomura, S. Nagamine, Intracellular recovery of gold by microbial reduction of AuCl 4 - ions using the anaerobic bacterium Shewanella algae, Hydrometallurgy. 81 (2006) 24–29. https://doi.org/10.1016/j.hydromet.2005.09.006.

[3] U. Shedbalkar, R. Singh, S. Wadhwani, S. Gaidhani, B.A. Chopade, Microbial synthesis of gold nanoparticles: Current status and future prospects, Adv. Colloid Interface Sci. 209 (2014) 40–48. https://doi.org/10.1016/j.cis.2013.12.011.

[4] M.F. Lengke, M.E. Fleet, G. Southam, Morphology of gold nanoparticles synthesized by filamentous cyanobacteria from gold(I)-Thiosulfate and gold(III)-chloride complexes, Langmuir. 22 (2006) 2780–2787. https://doi.org/10.1021/la052652c.

[5] R. Brayner, H. Barberousse, M. Hemadi, C. Djedjat, C. Yéprémian, T. Coradin, J. Livage, F. Fiévet, A. Couté, Cyanobacteria as bioreactors for the synthesis of Au, Ag, Pd, and Pt nanoparticles via an enzyme-mediated route, J. Nanosci. Nanotechnol. 7 (2007) 2696–2708. https://doi.org/10.1166/jnn.2007.600.

[6] K. Govindaraju, S.K. Basha, V.G. Kumar, G. Singaravelu, Silver, gold and bimetallic nanoparticles production using single-cell protein (Spirulina platensis) Geitler, J. Mater. Sci. 43 (2008) 5115–5122. https://doi.org/10.1007/s10853-008-2745-4.

[7] N. Chakraborty, A. Banerjee, S. Lahiri, A. Panda, A.N. Ghosh, R. Pal, Biorecovery of gold using cyanobacteria and an eukaryotic alga with special reference to nanogold formation - A novel phenomenon, J. Appl. Phycol. 21 (2009) 145–152. https://doi.org/10.1007/s10811-008-9343-3.

[8] M. Lengke, G. Southam, Bioaccumulation of gold by sulfate-reducing bacteria cultured in the presence of gold(I)-thiosulfate complex, Geochim. Cosmochim. Acta. 70 (2006) 3646–3661. https://doi.org/10.1016/j.gca.2006.04.018.

[9] Y. Feng, Y. Yu, Y. Wang, X. Lin, Biosorption and bioreduction of trivalent aurum by

photosynthetic bacteria Rhodobacter capsulatus, Curr. Microbiol. 55 (2007) 402–408. https://doi.org/10.1007/s00284-007-9007-6.

[10] S. He, Z. Guo, Y. Zhang, S. Zhang, J. Wang, N. Gu, Biosynthesis of gold nanoparticles using the bacteria Rhodopseudomonas capsulata, Mater. Lett. 61 (2007) 3984–3987. https://doi.org/10.1016/j.matlet.2007.01.018.

[11] N.J. Creamer, I.P. Mikheenko, P. Yong, K. Deplanche, D. Sanyahumbi, J. Wood, K. Pollmann, M. Merroun, S. Selenska-Pobell, L.E. Macaskie, Novel supported Pd hydrogenation bionanocatalyst for hybrid homogeneous/heterogeneous catalysis, Catal. Today. 128 (2007) 80–87. https://doi.org/10.1016/j.cattod.2007.04.014.

[12] L. Du, H. Jiang, X. Liu, E. Wang, Biosynthesis of gold nanoparticles assisted by Escherichia coli DH5α and its application on direct electrochemistry of hemoglobin, Electrochem. Commun. 9 (2007) 1165–1170. https://doi.org/10.1016/j.elecom.2007.01.007.

[13] M.I. Husseiny, M.A. El-Aziz, Y. Badr, M.A. Mahmoud, Biosynthesis of gold nanoparticles using Pseudomonas aeruginosa, Spectrochim. Acta Part A Mol. Biomol. Spectrosc. 67 (2007) 1003–1006. https://doi.org/10.1016/j.saa.2006.09.028.

[14] A. Mewada, G. Oza, S. Pandey, M. Sharon, W. Ambernath, Extracellular Biosynthesis of Gold Nanoparticles Using Pseudomonas denitiricans and Comprehending its Stability, J. Microbiol. Biotechnol. Res. 2 (2012) 493–499.

[15] Y. Konishi, T. Nomura, T. Tskukiyama, N. Saitoh, Microbial Preparation of Gold Nanoparticles by Anaerobic Bacterium, Trans Mater Res Soc Jpn. 2343 (2004) 2341–2343. http://sciencelinks.jp/j-east/article/200423/000020042304A0742483.php.

[16] A.K. Suresh, D.A. Pelletier, W. Wang, M.L. Broich, J.W. Moon, B. Gu, D.P. Allison, D.C. Joy, T.J. Phelps, M.J. Doktycz, Biofabrication of discrete spherical gold nanoparticles using the metal-reducing bacterium Shewanella oneidensis, Acta Biomater. 7 (2011) 2148–2152. https://doi.org/10.1016/j.actbio.2011.01.023.

[17] N. Sharma, A.K. Pinnaka, M. Raje, A. FNU, M.S. Bhattacharyya, A.R. Choudhury, Exploitation of marine bacteria for production of gold nanoparticles, Microb. Cell Fact. 11 (2012) 1–6. https://doi.org/10.1186/1475-2859-11-86.

[18] L. Wen, Z. Lin, P. Gu, J. Zhou, B. Yao, G. Chen, J. Fu, Extracellular biosynthesis of monodispersed gold nanoparticles by a SAM capping route, J. Nanoparticle Res. 11 (2009) 279–288. https://doi.org/10.1007/s11051-008-9378-z.

[19] A. Mohammed Fayaz, M. Girilal, M. Rahman, R. Venkatesan, P.T. Kalaichelvan, Biosynthesis of silver and gold nanoparticles using thermophilic bacterium Geobacillus stearothermophilus, Process Biochem. 46 (2011) 1958–1962. https://doi.org/10.1016/j.procbio.2011.07.003.

[20] S. Krishnamurthy, Y.S. Yun, Recovery of microbially synthesized gold nanoparticles using sodium citrate and detergents, Chem. Eng. J. 214 (2013) 253–261. https://doi.org/10.1016/j.cej.2012.10.028.

[21] K. Kalishwaralal, V. Deepak, S.B. Ram Kumar Pandian, M. Kottaisamy, S. BarathManiKanth, B. Kartikeyan, S. Gurunathan, Biosynthesis of silver and gold nanoparticles using Brevibacterium casei, Colloids Surfaces B Biointerfaces. 77 (2010) 257–262. https://doi.org/10.1016/j.colsurfb.2010.02.007.

[22] A. Ahmad, S. Senapati, M.I. Khan, R. Kumar, M. Sastry, Ahmad, S.Senapati, M.Islam Kham, R.Kumar, M.Sastry, Extracecullular biosynthesis of monodisperse gold nanoparticles by a novel extremophilic Actinomycete, Thermomonosporasp, ACS symp. Ser. Am. Chem. Soc. 19 (2013) 3550 – 3553, 19 (2003) 3550–3553.

[23] A. Ahmad, S. Senapati, M.I. Khan, R. Kumar, R. Ramani, V. Srinivas, M. Sastry, Intracellular synthesis of gold nanoparticles by a novel alkalotolerant actinomycete, Rhodococcus species, Nanotechnology. 14 (2003) 824–828. https://doi.org/10.1088/0957-4484/14/7/323.

[24] T. Kalabegishvili, Synthesis of gold nanoparticles by some strains of Arthrobacter genera, J. Mater. …. 2 (2012) 164–173. http://www.researchgate.net/profile/Marina_Frontasyeva2/publication/233919170_Synthesis_of_gold_nanoparticles_by_some_strains_of_Arthrobacter_genera/links/02bfe5108f97a04fd0000000.pdf.

[25] K. Deplanche, L.E. Macaskie, Biorecovery of gold by Escherichia coli and Desulfovibrio desulfuricans, Biotechnol. Bioeng. 99 (2008) 1055–1064. https://doi.org/10.1002/bit.21688.

[26] S. Wang, K. Qian, X. Bi, W. Huang, Influence of speciation of aqueous HAuCl4 on the synthesis, structure, and property of Au colloids, J. Phys. Chem. C. 113 (2009) 6505–6510. https://doi.org/10.1021/jp811296m.

[27] J. Varia, S.S. Martínez, S.V. Orta, S. Bull, S. Roy, Bioelectrochemical metal remediation and recovery of Au3+, Co2+ and Fe3+ metal ions, Electrochim. Acta. 95 (2013) 125–131. https://doi.org/10.1016/j.electacta.2013.02.051.

[28] N. Zhu, Y. Cao, C. Shi, P. Wu, H. Ma, Biorecovery of gold as nanoparticles and its catalytic activities for p-nitrophenol degradation, Environ. Sci. Pollut. Res. 23 (2016) 7627–7638. https://doi.org/10.1007/s11356-015-6033-y.

[29] C. Flores, R. O`Keefe, Gold recovery from organic solvents using galvanic stripping, in: Minerals, Metals and Materials Society, Warrendale, PA (United States), United States, 1995. https://www.osti.gov/servlets/purl/78163.

[30] S. De Corte, T. Hennebel, S. Verschuere, C. Cuvelier, W. Verstraete, N. Boon, Gold nanoparticle formation using Shewanella oneidensis: A fast biosorption and slow reduction process, J. Chem. Technol. Biotechnol. 86 (2011) 547–553. https://doi.org/10.1002/jctb.2549.

[31] J. Varia, A. Zegeye, S. Roy, S. Yahaya, S. Bull, Shewanella putrefaciens for the remediation of Au3+, Co2+ and Fe3+ metal ions from aqueous systems, Biochem. Eng. J. 85 (2014) 101–109. https://doi.org/10.1016/j.bej.2014.02.002.

[32] F. Reith, B. Etschmann, C. Grosse, H. Moors, M. a Benotmane, P. Monsieurs, G. Grass, C. Doonan, S. Vogt, B. Lai, G. Martinez-Criado, G.N. George, D.H. Nies, M. Mergeay, A. Pring, G. Southam, J. Brugger, Mechanisms of gold biomineralization in the bacterium Cupriavidus metallidurans, Proc. Natl. Acad. Sci. 106 (2009) 17757–17762. https://doi.org/10.1073/pnas.0904583106.
